# Tuning carbon quantum dot optics through nitrogen surface chemistry

**DOI:** 10.1039/d6ra07516a

**Published:** 2026-10-14

**Authors:** Shweta Dua, Pawan Kumar, Sakshi Kapoor, Manoj Kumar Khanna, Geeta Bhatt

**Affiliations:** a Bhaskaracharya College of Applied Sciences, University of Delhi Delhi India; b Ramjas College, University of Delhi Delhi India; c Indian Institute of Technology Delhi India; d Department of Electronic Science, University of Delhi South Campus Delhi India; e Non-Collegiate Women's Education Board, University of Delhi Delhi India geeta.bhatt@bcas.du.ac.in

## Abstract

Nitrogen-doped carbon quantum dots (N-CQDs) were synthesized *via* rapid microwave carbonization of citric acid monohydrate and urea at three CA : Urea mass ratios—2 : 1 (B1-CQD), 1 : 1 (B2-CQD), and 1 : 2 (B3-CQD) to systematically investigate how precursor stoichiometry influences their structural, compositional, and optical properties. XRD and Raman spectroscopy indicated semi-graphitic carbon structures with increasing structural disorder at higher urea content, together with additional crystalline reflections in B3-CQD attributed to reaction by-products. TEM revealed a decrease in particle size with increasing urea content, with B3-CQD exhibiting the smallest (2.95 ± 0.89 nm) and most monodisperse dots. EDX and XPS analyses showed a systematic increase in nitrogen content from 20.5% (B1-CQD) to 31.9% (B3-CQD) by EDX, while total oxygen remained nearly invariant (∼28–30%). The surface-to-bulk nitrogen ratio (SBNR), defined as the ratio of the XPS- to the EDX-derived nitrogen content, increased progressively from 34.1% to 82.4% with increasing urea content, approaching parity between the surface and bulk nitrogen contents; because the XPS sampling depth is comparable to the particle size, the SBNR is presented as a descriptive composition ratio. High-resolution N 1s XPS indicated a redistribution among nitrogen environments, from predominantly pyridinic/amine nitrogen in B1-CQD toward pyrrolic/amide nitrogen in B3-CQD, which XPS alone cannot fully resolve. UV-Vis absorption showed principal maxima at 342.7 nm (B1-CQD), 328.7 nm (B2-CQD), and 328.0 nm (B3-CQD), with an additional shoulder at 273–274 nm in B2-CQD and B3-CQD. Tauc analysis revealed a core-state (π → π*) gap of ∼5.4–5.5 eV together with sub-gap levels between 2.61 and 4.24 eV associated with nitrogen- and oxygen-containing groups. As particle size and nitrogen content vary together in this series, their individual contributions to the optical properties cannot be separated. Excitation-dependent photoluminescence was observed in all samples; under 360 nm excitation, the emission maxima were 438–442 nm, and B3-CQD showed a maximum at 370 nm under 320 nm excitation. Collectively, these findings show that precursor stoichiometry tunes the nitrogen content, surface chemistry, and optical properties of the filtered N-CQD dispersions, which may also contain co-produced molecular species, providing a structure–chemistry–property framework for rational CQD design.

## Abstract 2

Increasing the urea content of microwave-synthesized citric acid–urea carbon quantum dots raises their nitrogen content and surface-to-bulk nitrogen ratio, and introduces nitrogen- and oxygen-related sub-gap states that their emission follows.

## Graphical abstract

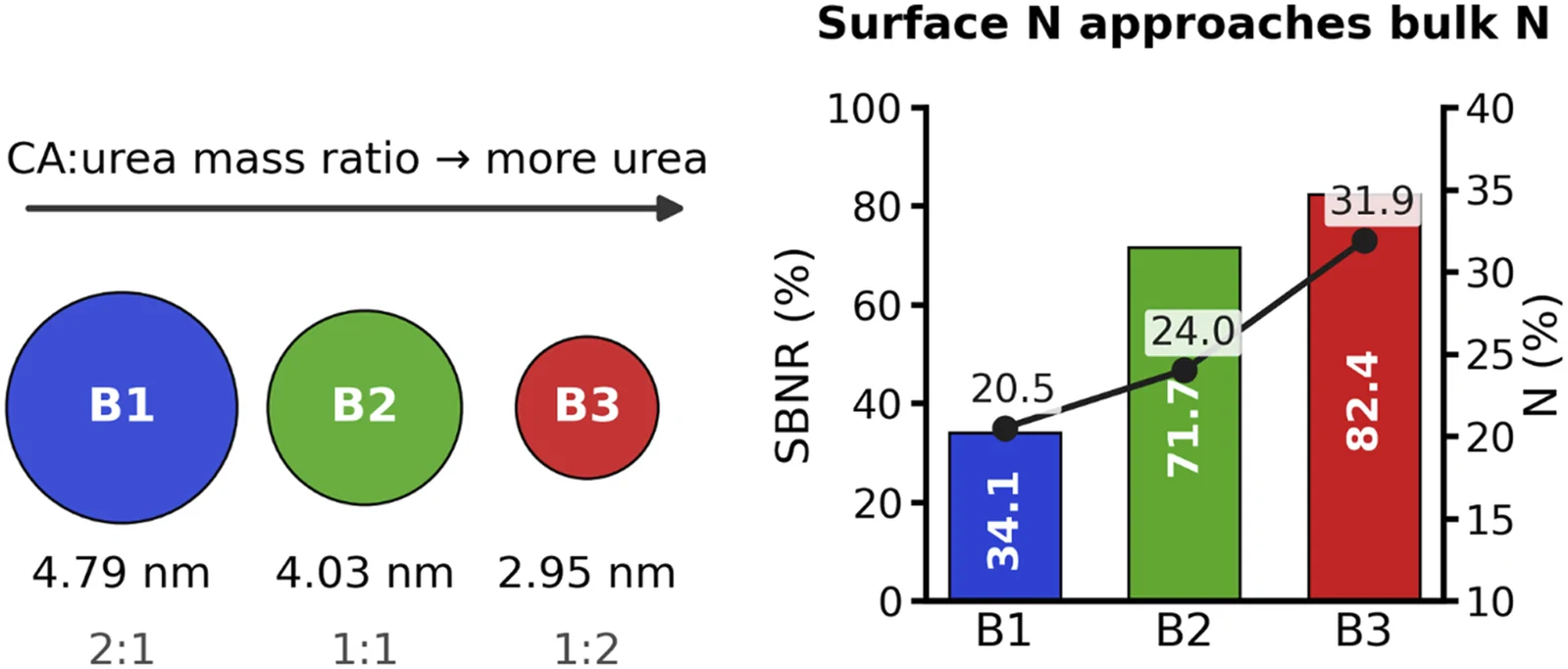

## 1. Introduction

Carbon quantum dots (CQDs) represent a prominent class of fluorescent nanomaterials, having been serendipitously discovered in 2004 during the purification of single-walled carbon nanotubes. Owing to their exceptional optical properties, low toxicity, excellent biocompatibility, chemical stability, and facile synthesis from abundant precursors, CQDs have attracted enormous research interest across diverse applications, including bio-imaging, fluorescence sensing, photocatalysis, light-emitting diodes, and solar energy harvesting.^1–5^

Among the various strategies employed to tailor the opto-electronic properties of CQDs, nitrogen doping has emerged as one of the most effective and widely adopted approaches. The incorporation of nitrogen heteroatoms into the carbon framework modifies the electronic structure through the introduction of new energy states within the bandgap, alters the surface chemistry by creating nitrogen-containing functional groups (pyridinic, pyrrolic, graphitic, and amino nitrogen), and enhances photoluminescence quantum yield through improved surface passivation.^5–10^

Microwave-assisted synthesis has gained considerable attention as a rapid, energy-efficient, and scalable route for CQD fabrication, enabling uniform volumetric heating that accelerates carbonization and doping to completion within minutes.^11–13^

Citric acid (CA) and urea are among the most widely employed and economical precursor combinations for the synthesis of N-CQDs due to their abundant availability, low cost, scalability, and high carbon, nitrogen, and oxygen content. It produces CQDs with tunable optical properties.^13,14^ The carbonization of CA yields a graphitic carbon core with abundant carboxyl and hydroxyl surface groups. At the same time, the thermal decomposition of urea releases reactive nitrogen species that are incorporated into the carbon framework as dopants and surface functional groups.^15–18^ The relative ratio of these two precursors fundamentally governs the degree of nitrogen doping, particle size, and optical properties of the resulting CQDs. Several earlier studies have varied the citric acid-to-urea ratio to change the CQD emission color and have used XPS to confirm nitrogen doping.^19–21^

However, one important question remains unresolved: which specific type of nitrogen bonding is actually responsible for changes in emission color? The precise photophysical role of the specific nitrogen-bonding configuration—pyridinic, pyrrolic, or graphitic—remains contested. One study found that photoluminescence becomes stronger when pyridinic nitrogen increases and weaker when pyrrolic nitrogen increases.^6^ Other studies instead link blue-shifted emission to amine nitrogen^9^ or to a combination of pyridinic and pyrrolic nitrogen together.^10^ In other words, no consistent picture exists yet. A second unresolved question concerns the origin of the optical bandgap itself. Both quantum confinement (particle size) and nitrogen doping (surface states) are known to influence the bandgap of CQDs. But which of these two effects actually dominates remains an open and actively debated question in the field.^22–24^

Beyond these two unresolved issues, another important limitation exists specifically for the citric acid–urea system. Although XPS and EDX are routinely used to determine nitrogen content, they are generally interpreted independently. Consequently, how the nitrogen content at the CQD surface—a region that strongly influences the electronic and optical properties—compares with the overall nitrogen content remains largely unexplored. To address this gap, the present study reports the surface-to-bulk nitrogen ratio (SBNR), defined as the ratio of nitrogen content determined by surface-sensitive XPS to that obtained by EDX, as a descriptive comparison between the surface and bulk nitrogen contents.

In this work, we present a systematic and comprehensive investigation of N-CQDs synthesized *via* microwave-assisted carbonization at three distinct CA : Urea mass ratios (2 : 1, 1 : 1, and 1 : 2). We aim to address these gaps simultaneously. A multi-evidence correlation between precursor stoichiometry and material properties is examined, together with the relative roles of nitrogen doping and particle-size effects in the optical behavior, recognizing that these two factors co-vary in this series. These include high-resolution XPS deconvolution of C 1s, N 1s, and O 1s spectra; a direct comparison between XPS (surface-sensitive) and EDX (bulk-sensitive) nitrogen contents *via* the SBNR to assess surface and bulk nitrogen content. This approach examines not only how much nitrogen is doped into the CQDs, but also how the nitrogen environments and the surface-to-bulk nitrogen distribution change, and how these changes relate to their optical behavior.

## 2. Experimental section

### 2.1 Materials

Citric acid monohydrate (C_6_H_8_O_7_·H_2_O, 99.5%) was procured from CDH Fine Chemicals, and urea (CH_4_N_2_O, 99.5%) from HiMedia Laboratories Pvt. Ltd Acetone (99.5%) and isopropyl alcohol (99.5%) were purchased from Sigma-Aldrich. All reagents were analytical grade and used as received without further purification.

All glassware used in the experiments was thoroughly cleaned with a laboratory detergent solution, rinsed with distilled water, and finally washed with acetone before use. All experimental procedures were conducted under ambient laboratory conditions.

### 2.2 Synthesis of N-doped carbon quantum dots

N-CQDs were synthesized through microwave radiation using citric acid and urea at three CA : Urea mass ratios 2 : 1, 1 : 1, and 1 : 2 (molar ratios 1 : 1.75, 1 : 3.50 and 1 : 7.00, calculated with molar masses of 210.14 g mol^−1^ for citric acid monohydrate and 60.06 g mol^−1^ for urea)—as summarized in Table 1. For each composition, precursors were dissolved in 20 mL of distilled water and magnetically stirred for 30 min (UTech digital stirrer, India). The homogeneous solutions were subjected to microwave carbonization (LG MH2046HB, India) at 700 W for 300 s, yielding solid carbonized products. After cooling, 0.1 g of each product was redispersed in 10 mL of distilled water, followed by ultrasonication for 1800 s (Citizen DSA, India), and filtered through 0.22 µm polytetrafluoroethylene (PTFE) syringe filters to yield filtered CQD stock dispersions (B1-CQD, B2-CQD, B3-CQD). Filtration removes insoluble particulates but not dissolved molecular species, so the filtered dispersions and the powders obtained by drying them may contain co-produced molecular species in addition to the CQDs. For studies requiring powdered CQDs, the filtered stock dispersions were dried in a hot-air oven at 40 °C for 12 h to allow controlled evaporation of water. The dried CQDs were then gently ground using a mortar and pestle to obtain fine, uniform CQD powder suitable for further analysis.

**Table 1:** Precursor compositions for N-doped CQD synthesis

Sample	CA : Urea (Mass ratio)	CA (g)	Urea (g)	CA : Urea (molar ratio)
B1-CQD	2 : 1	2	1	1 : 1.75
B2-CQD	1 : 1	1	1	1 : 3.50
B3-CQD	1 : 2	1	2	1 : 7.00

### 2.3 Characterization

XRD patterns were recorded by an X-ray diffractometer (Bruker-AXS-D8 Discover, America), which uses Cu Kα radiation (*λ* = 0.1542 nm) over 2*θ* = 10–70°. Crystallite domain sizes were estimated with the Scherrer equation (*K* = 0.9); instrumental broadening was not corrected, so the values are lower bounds. Raman spectra were recorded using a Raman spectrometer (Witec confocal microprobe, Oxford Instruments, Germany) with 532 nm laser excitation. Spectra were baseline-corrected with a linear baseline between 700 and 2050 cm^−1^, and the D and G bands were fitted with Lorentzian profiles; *I*_D_/*I*_G_ is reported as the ratio of integrated band areas. The CQD images were obtained by HR-TEM (JEOL, JEM-F200, Japan) at 200 kV, and particle sizes were determined by Laplacian-of-Gaussian blob detection implemented in scikit-image on fields recorded with 50 and 100 nm scale bars, with each image calibrated to its scale bar. Only clearly resolved, non-aggregated particles exceeding a uniform detection threshold, applied identically to all fields, were counted; aggregates and edge-truncated particles were excluded. EDX elemental analysis was performed by FESEM (TESCAN, Magna LMU, Czech Republic); spectra were recorded from a single field for each sample (632 µm image width, 15 kV, ∼90 s acquisition) and quantified with the “all elements (normalized)” routine. Elemental analysis was carried out using an XPS Spectrophotometer (Kratos Analytical Ltd-AXIS Supra, UK) having a monochromatic X-ray source (Al Kα). Spectra were charge-referenced to the C–C/C=C component of C 1 s at 284.4 eV and fitted with Gaussian components after Tougaard background subtraction; after fitting, the C–C/C=C component was located at 284.3–284.5 eV, within 0.1 eV of the reference value. Atomic percentages were obtained by dividing peak areas by relative sensitivity factors (RSFs) of 1.00 (C 1s), 1.80 (N 1s), and 2.93 (O 1s). FTIR spectra were recorded in transmittance mode (4000–500 cm^−1^) using an FTIR spectrophotometer (Bruker Alpha-E, Germany). UV-Vis absorption spectra were recorded using a double-beam UV-Vis spectrophotometer (PerkinElmer Lambda 35, USA) over the wavelength range of 200–700 nm; for these measurements, 50 µL of stock dispersion was diluted into 15 mL of distilled water (final concentration 0.0332 mg mL^−1^). Tauc plots were constructed as (*αhν*)^2^*vs. hν* to determine the direct bandgap. PL spectra of all three samples were recorded at the same concentration and under identical instrumental conditions. The excitation wavelengths were varied from 280 to 420 nm from an Upconversion and downconversion fluorescence spectrometer (Horiba Scientific, Model QM-8450-11, US) using excitation and emission monochromators without an additional emission filter; each emission spectrum was recorded only up to wavelengths below twice the excitation wavelength to exclude second-order diffraction. PL intensities are relative values recorded under identical conditions; quantum yields were not determined.

## 3. Results and discussion

The structural properties of B1-CQD, B2-CQD, and B3-CQD were investigated using XRD and Raman spectroscopy. XRD probes the long-range interlayer stacking order of the graphitic layers, while Raman spectroscopy examines the short-range in-plane structural quality within each graphitic layer. Thus, together, they deliver a complete structural picture of the synthesized CQDs.

The normalized XRD patterns of B1-CQD, B2-CQD, and B3-CQD are presented in Fig. 1. All three samples exhibit a broad diffraction peak centered at 2*θ* ≈ 27.3°, corresponding to (002) reflection plane of graphitic carbon, with an interlayer *d*-spacing (∼0.326 nm) slightly smaller than that of ideal graphite (0.335 nm). At the same time, many carbon dot systems exhibit *d*-spacings larger than those of graphite due to turbostratic disorder and bulky surface functional groups.^25,26^ In the present samples, however, the smaller *d*-spacing suggests a different structural origin: heteroatom-mediated cross-linking—particularly C–N–C and C–O–C bridges between adjacent graphitic layers—which can pull the layers closer together than the simple van der Waals stacking seen in pure graphite.^27^

**Fig. 1:**
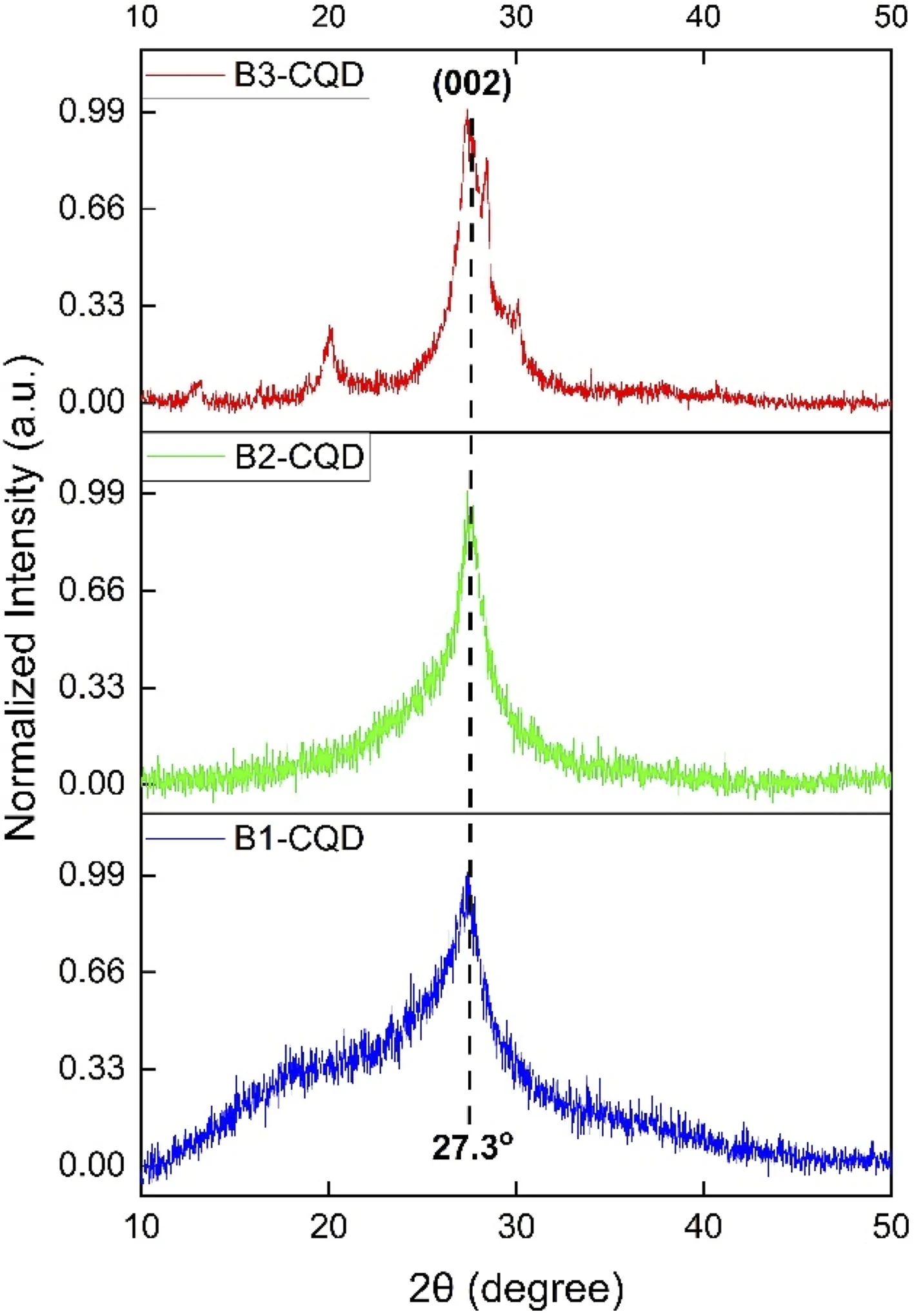
XRD patterns of B1-CQD (blue), B2-CQD (green), and B3-CQD (red) showing the (002) diffraction peak at 2*θ* ≈ 27.3°. B3-CQD shows additional reflections (∼20.1°, 28.4°, and 29.7°) attributed to crystalline by-products of the citric acid–urea reaction.

The structural evolution and layer-to-layer stacking characteristics of the synthesized CQDs were evaluated using XRD analysis, as shown in the normalized patterns in Fig. 1. All three samples exhibit a prominent reflection centered at approximately 27.3°, corresponding to the (002) plane of graphitic carbon structures. The structural refinement from B1-CQD to B3-CQD was evaluated using peak broadening (Full Width at Half Maximum, FWHM) and line-shape characteristics. The (002) reflection of B1-CQD is broad and diffuse (FWHM 4.22°), characteristic of a predominantly disordered, turbostratic framework,^28,29^ whereas that of B2-CQD is narrower (FWHM 2.73°), indicating larger ordered domains (Scherrer sizes 1.9 and 3.0 nm; Table S1).^30^ The apparently narrower reflection of B3-CQD is not interpreted in this way because it overlaps with the reflections of the crystalline by-product discussed below.

B3-CQD additionally shows reflections at around 20.05°, 28.41°, and 29.7°, which are attributed to crystalline by-products of the citric acid–urea reaction retained in the filtered product.^16,19^ XRD peak fitting of the (002)-type reflection and the additional reflections of B3-CQD, with Scherrer domain sizes, is shown in Table S1. Scherrer analysis of the (002)-type reflection yields domain sizes of 1.9 and 3.0 nm for B1-CQD and B2-CQD (lower bounds; Table S1), which are smaller than their TEM mean diameters, as expected. For B3-CQD, the (002)-type reflection overlaps the by-product reflections, so its apparent Scherrer size (4.3 nm) does not measure the CQD domain size and is not interpreted. In contrast, the narrow 28.41° reflection of B3-CQD corresponds to a domain of about 28–36 nm, which cannot arise from the CQDs and indicates a separate crystalline phase. The relatively higher degree of interlayer ordering in the present CQDs, compared with most reports in the literature,^25^ is consistent with the well-defined semi-graphitic character indicated by Raman spectroscopy.

Raman spectra of all three CQD samples are shown in Fig. 2, revealing two characteristic carbon bands: (i) D-band at 1354–1355 cm^−1^ the “Defect band” arising from the defect-activated breathing mode of sp^2^ carbon rings, to which nitrogen incorporation, surface functional groups and edge defects can all contribute^31^ and (ii) the G-band at 1572–1578 cm^−1^ the “Graphitic band” arising from the in-plane stretching vibration of C=C bonds in the sp^2^-hybridized carbon (core) of the CQDs. Importantly, given the very small particle dimensions of the CQDs (2.95–4.79 nm) and their correspondingly high surface-to-volume ratio, it is reasonable to expect that a substantial fraction of the D-band intensity in these materials originates from surface rather than bulk core defects, consistent with the well-established origin of the D-band in edge and defect sites of the graphitic lattice.^31^ This provides a plausible, though indirect, link between D-band intensity and surface nitrogen content, which is compared with bulk nitrogen content *via* the SBNR discussed in the EDX & XPS section. Band positions and *I*_D_/*I*_G_ (integrated area) ratios were obtained after linear baseline subtraction and Lorentzian fitting (Table 2); the fits are shown in Fig. S1.

**Fig. 2:**
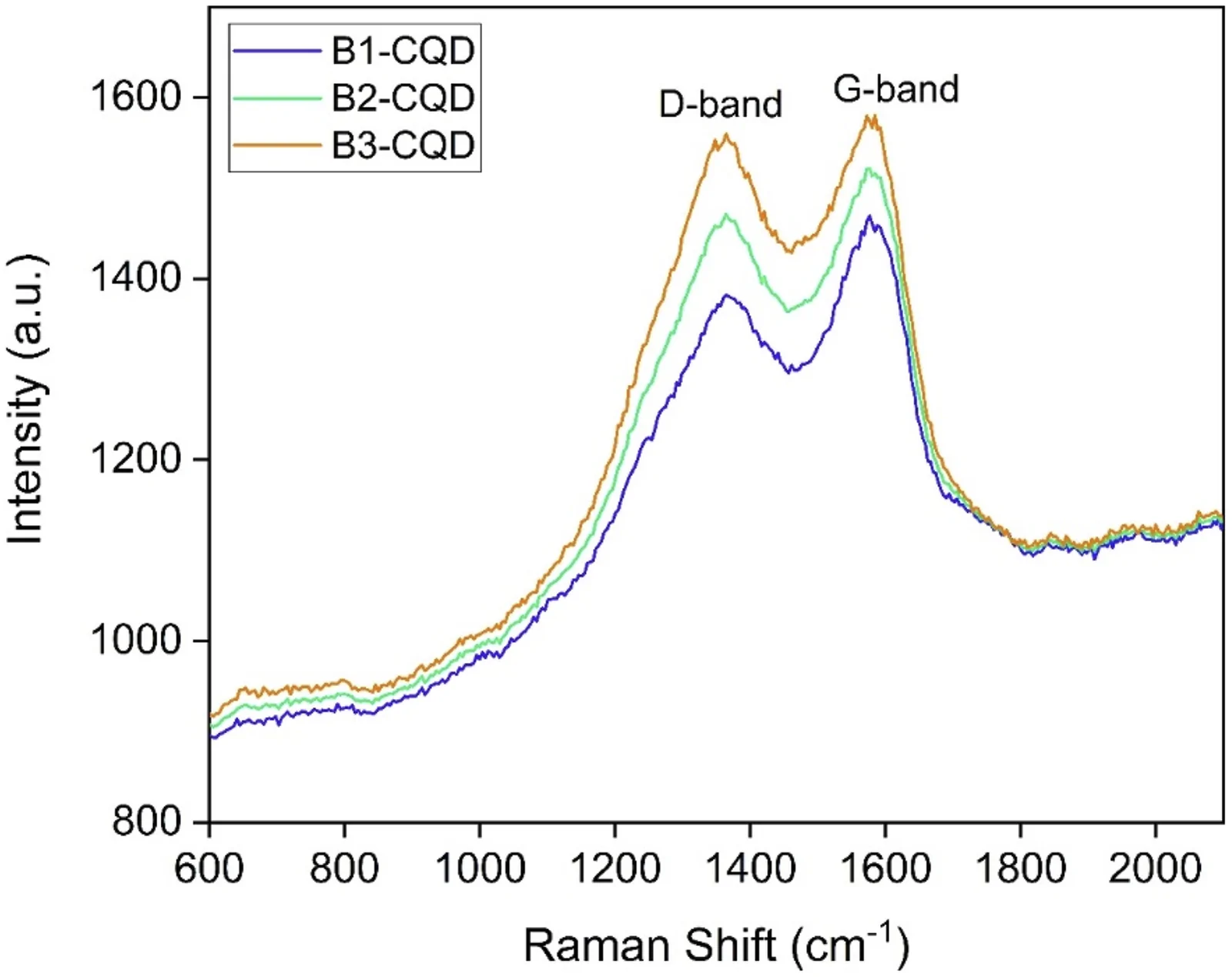
Raman spectra of B1-CQD (blue), B2-CQD (green), and B3-CQD (Red) showing the D and G bands.

**Table 2:** Raman band parameters and *I*_D_/*I*_G_ ratios (integrated area)

Sample	D-band (cm^−1^)	G-band (cm^−1^)	*I*_D_/*I*_G_ (integrated area)
B1-CQD	1355	1578	1.99
B2-CQD	1354	1575	2.15
B3-CQD	1354	1572	2.26

A systematic increase in *I*_D_/*I*_G_ ratio from 1.99 (B1-CQD) to 2.15 (B2-CQD) to 2.26 (B3-CQD) is observed with increasing urea content (Table 2). This is consistent with the formation of more defect sites within the carbon framework at higher nitrogen incorporation. This increasing disorder trend is consistent with the increase in nitrogen content and in the SBNR (XPS section) as urea content increases.

The *I*_D_/*I*_G_ values of all three samples (1.99–2.26) indicate the semi-graphitic, defect-enriched carbon structure of the synthesized CQDs. This intermediate structural character, neither fully graphitic nor fully amorphous, is particularly desirable for photoluminescence, as the nitrogen-induced surface defect sites can act as emissive centers that trap and radiatively release photo-excited electrons as visible light.^17^

TEM analysis confirmed the formation of quasi-spherical, well-dispersed CQDs for all three compositions (Fig. 3a–c). Size distribution histograms (Fig. 3d–f), plotted as measured from fields recorded at 50 and 100 nm scale bars (particle diameter determined by Laplacian of Gaussian detection),^32,33^ yielded mean diameters of 4.79 ± 1.74 nm (B1-CQD, *n* = 35), 4.03 ± 1.65 nm (B2-CQD, *n* = 26), and 2.95 ± 0.89 nm (B3-CQD, *n* = 521), as summarized in Table 3. Fields recorded at the 200 nm scale bar were not used because they do not resolve the smallest particles. Representative annotated TEM fields that mark the counted particles are shown in Fig. S2.

**Fig. 3:**
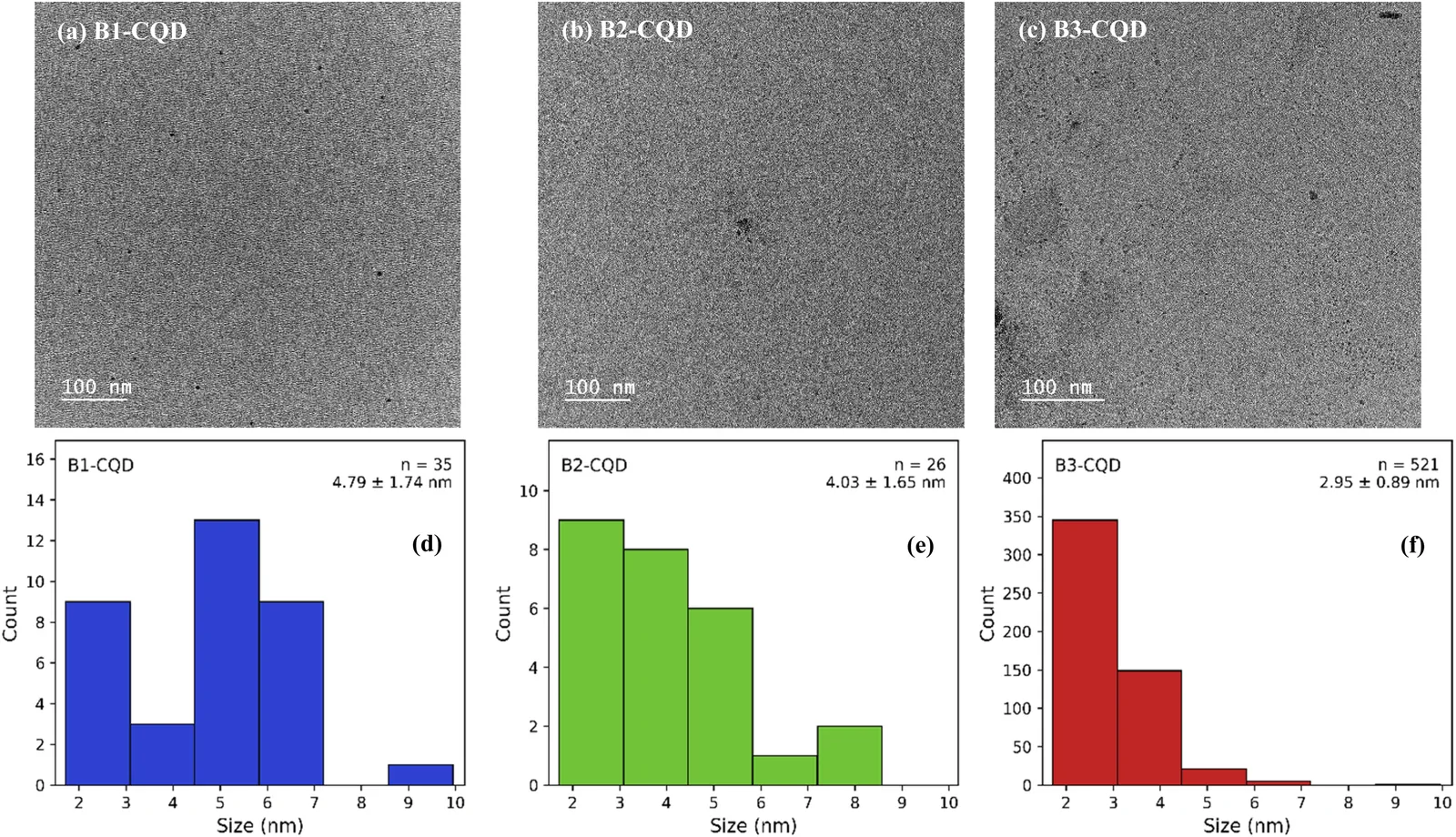
TEM images of (a) B1-CQD, (b) B2-CQD, and (c) B3-CQD showing quasi-spherical nanoparticles (100 nm scale bar). Corresponding particle size distribution histograms, plotted as measured from fields recorded at 50 and 100 nm scale bars, for (d) B1-CQD (4.79 ± 1.74 nm, *n* = 35), (e) B2-CQD (4.03 ± 1.65 nm, *n* = 26), and (f) B3-CQD (2.95 ± 0.89 nm, *n* = 521) (mean ± SD). Histogram bin width: 1.37 nm.

**Table 3:** Particle size distribution parameters from TEM analysis

Sample	Mean size (nm)	Std. deviation (nm)	Size range (nm)	*n* (particles)
B1-CQD	4.79	±1.74	2.4–9.3	35
B2-CQD	4.03	±1.65	2.4–7.9	26
B3-CQD	2.95	±0.89	2.4–9.3	521

The decrease in particle size with increasing urea content is attributed to the role of urea: at the highest urea content (B3-CQD), excess urea caps nascent CQD nuclei during carbonization, restricting growth and yielding the smallest, most monodisperse dots (2.95 ± 0.89 nm).

EDX elemental analysis (Fig. S3) showed carbon, nitrogen, and oxygen as the principal elements, together with a minor aluminium signal (∼0.4%, similar in all samples) that was not detected by XPS and is attributed to the aluminium sample stub (Table S2). As standardless EDX quantification of light elements is semi-quantitative,^34^ the EDX values tend across the series rather than as absolute compositions.

Nitrogen content increased systematically from 20.5 to 31.9% (B1-CQD to B3-CQD), representing a 55.6% relative enhancement, while carbon content decreased from 50.7 to 38.3% and oxygen remained essentially invariant (∼28–30%) (Table 4). The EDX N/C ratio increased from 0.404 to 0.833, serving as a measure of the degree of nitrogen incorporation. The total oxygen content is similar across the series; whether the oxygen speciation also changes was examined by XPS.

**Table 4:** Elemental composition (EDX) and Raman disorder parameter. EDX values (%) are from one spectrum per sample; their statistical uncertainties are given in Table S2

Sample	C (%)	N (%)	O (%)	N/C ratio (EDX)	*I*_D_/*I*_G_ (area)
B1-CQD	50.7	20.5	28.8	0.404	1.99
B2-CQD	47.7	24.0	28.3	0.503	2.15
B3-CQD	38.3	31.9	29.8	0.833	2.26

XPS survey spectra (Fig. S4) confirmed the exclusive presence of C 1s (∼284 eV), N 1s (∼400 eV), and O 1s (∼532 eV) peaks, with no detectable impurity signals across 0–900 eV within the XPS sampling depth. The N 1s peak intensity increased substantially while C 1s decreased from B1-CQD to B3-CQD, consistent with the EDX trend; quantitative XPS compositions were obtained from RSF-corrected peak areas (Table S3).^35^

Quantitative analysis using peak area/relative sensitivity factor (RSF) values (Table S3) revealed that the nitrogen atomic concentration determined by XPS increased from 7.0% for B1-CQD to 17.2% for B2-CQD and 26.3% for B3-CQD. To compare the surface and bulk nitrogen contents, the surface-to-bulk nitrogen ratio (SBNR) was defined as the ratio of nitrogen content measured by XPS to that determined by EDX. The SBNR increased progressively from 34.1% for B1-CQD to 71.7% for B2-CQD and 82.4% for B3-CQD, approaching parity between the surface and bulk nitrogen contents as urea concentration increased (Table 5). This trend, together with the progressive redistribution of nitrogen from predominantly pyridinic to pyrrolic/amide configurations revealed by N 1s analysis, suggests that increasing urea concentration preferentially promotes the incorporation of nitrogen into surface-associated functional environments. Although XPS and EDX cannot directly resolve the spatial distribution of nitrogen within individual nanoparticles, the SBNR is presented as a descriptive composition ratio without mechanistic interpretation: the XPS sampling depth is comparable to the particle size, and adventitious hydrocarbon contamination would also lower the ratio. Definitive confirmation of the nitrogen distribution, however, would require depth-resolved or spatially resolved techniques.

**Table 5:** Surface-to-bulk nitrogen ratio (SBNR)

Sample	EDX N (%)	XPS N 1s (%)	SBNR (%)
B1-CQD	20.5	7.0	34.1
B2-CQD	24.0	17.2	71.7
B3-CQD	31.9	26.3	82.4

Prior studies of citric acid/urea-derived carbon dots have examined how nitrogen configuration (pyridinic, pyrrolic, graphitic) relates to particle size and photoluminescence.^6,37–39^ Comparing surface-sensitive with bulk composition is an established practice; to our knowledge, however, a direct ratio of XPS-derived to EDX-derived nitrogen content has not been reported for this precursor system. The XPS sampling depth is a well-established limitation for surface-composition measurements on nanoscale carbon dots,^27^ and is directly relevant to the particle sizes studied here.

High-resolution C 1s spectra (Fig. 4) were deconvoluted into C–C/C=C (∼284.3–284.5 eV), C–N/C–O (∼285.8–286.6 eV), C=O/N–C=O (∼287.6–287.9 eV), O–C=O and/or N-containing C=O (∼289.1–289.4 eV), and, in the fits of B2-CQD and B3-CQD, a π → π* shake-up satellite (∼290.8 eV). The π → π* shake-up feature is characteristic of aromatic sp^2^-conjugated carbon domains and enhanced π-electron delocalization.^36^ The ∼289 eV component cannot be assigned solely to O–C=O because the required oxygen content would exceed the measured value; it may include a nitrogen-containing carbonyl carbon, and its assignment is therefore tentative. Notably, B2-CQD and B3-CQD also exhibit additional sub-gap optical transitions compared to B1-CQD (Table 6). These observations are consistent with a contribution of conjugated domains and nitrogen-containing surface states to the electronic structure and optical response of the CQDs.

**Fig. 4:**
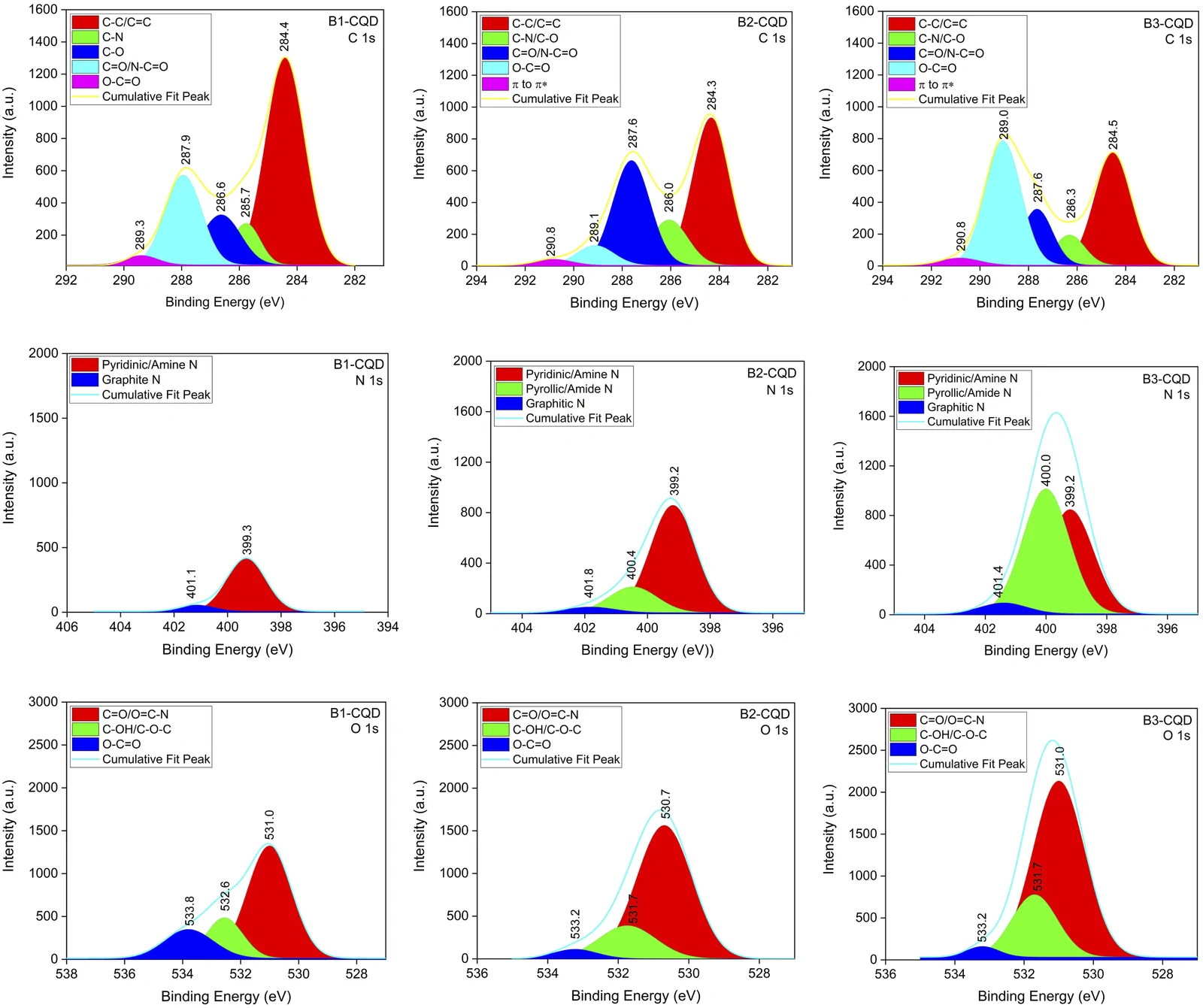
Individual high-resolution deconvoluted XPS spectra: C 1s, N 1s, and O 1s for B1-CQD, B2-CQD & B3-CQD. Spectra were charge-referenced to the C–C/C=C component at 284.4 eV and fitted with Gaussian components after background subtraction by Tougaard; the fitted C–C/C=C positions lie at 284.3–284.5 eV. The ∼289 eV C 1s component is assigned tentatively to O–C=O and/or N-containing C=O. The fits to the raw data points and residuals are shown in Fig. S5, and the fitted parameters with their uncertainties are listed in Table S4.

**Table 6:** Optical transition energies from Tauc plot analysis

Sample	Core-state gap, π → π^a^ (eV)	*R* ^2^ (core-state fit)	Sub-gap onsets (eV)^b^
B1-CQD	5.52	0.996	3.01
B2-CQD	5.50	0.993	2.61; 3.51; 4.23
B3-CQD	5.43^a^	0.994	2.67; 3.55; 4.24

a Indicative only: at the common measurement concentration (0.0332 mg mL^−1^), the absorbance in the fitted region (1.57–3.28) exceeded the reliable range of the spectrophotometer.

b Onsets at 3.0–3.6 eV belong to the ∼330–340 nm band and are attributed to n → π* transitions of C=O/C=N groups.^47,48^ Onsets at 2.6–2.7 eV belong to the weak band above 400 nm and are attributed to lone-pair surface states.^48^ Onsets at ∼4.2 eV belong to the 273–274 nm shoulder and are tentatively attributed to π → π* transitions of C=N/C=O-containing conjugated rings.^19,48^ In citric acid–urea products, the ∼330 and ∼420 nm bands are also associated with citrazinic-acid-type species.^19,46^

The high-resolution N 1s spectra (Fig. 4) indicate a redistribution among nitrogen environments across the three CQD samples, which XPS alone cannot fully resolve, because pyridinic and amine nitrogen, and pyrrolic and amide nitrogen, overlap in binding energy. In B1-CQD, nitrogen is predominantly present as pyridinic/amine species (399.3 eV, ∼90%), accompanied by a minor graphitic nitrogen contribution (401.1 eV, ∼10%). At the same time, a pyrrolic/amide component cannot be excluded within the broad envelope. Upon increasing the urea content (B2-CQD), a pyrrolic/amide component (400.5 eV) is required in the fit, accompanied by a lower relative pyridinic/amine fraction (Table S4). In B3-CQD, the pyrrolic/amide component (400.0 eV) becomes comparable to or larger than the pyridinic/amine component, although both carry large fitting uncertainties (Table S4). This redistribution among nitrogen environments parallels the evolution of the optical properties across the series. Additional nitrogen introduced by increasing urea concentration appears to be accommodated largely in pyrrolic and amide configurations, a trend consistent with the precursor-dependent evolution of nitrogen functionalities reported for N-doped carbon quantum dots. Concurrently, the SBNR increases (Table 5), indicating that the surface nitrogen content approaches the bulk nitrogen content with increasing urea concentration. Collectively, the redistribution of nitrogen environments may modify the surface electronic structure and defect states, contributing to the emergence of additional sub-gap optical transitions.^37,38^

High-resolution O 1s spectra (Fig. 4) were deconvoluted into three oxygen-containing components assigned to C=O/O=C–N (∼530.7–531.0 eV), C–OH/C–O–C (∼531.7–532.8 eV), and O–C=O (∼533.2–533.9 eV), consistent with O 1s assignments commonly reported for citric acid-derived and N-doped carbon quantum dots.^38,40^ The fitted fractions differ among samples; for example, the O–C=O component accounts for 16.5%, 4.3%, and 1.4% of the O 1s area in B1-CQD, B2-CQD, and B3-CQD, respectively. However, the components of B2-CQD and B3-CQD are not independently resolved and carry large fitting uncertainties (Table S4). Thus, although the total oxygen content is similar across the series, the oxygen speciation also varies, so the changes in oxygen-containing groups may contribute to the observed structural and optical changes alongside nitrogen.

FTIR spectra (Fig. S6) revealed rich surface functional group chemistry in all three samples. The broad absorption at 3200–3500 cm^−1^ (O–H/N–H stretch) progressively intensifies from B1-CQD to B3-CQD, consistent with an increasing N–H contribution. C–H stretching feature near 2950 cm^−1^ is most prominent in B1-CQD and diminishes with increasing urea content. The intense band at ∼1700–1720 cm^−1^ (C=O stretch of –COOH and amide groups) remains invariant across samples, in agreement with stable oxygen content. Bands at ∼1580–1630 cm^−1^ (C=C/C=N stretch) grow increasingly significant in B2-CQD and B3-CQD, corroborating Raman G-band observations. The multiplicity of C–N, C–O, and N–H bands in the 1000–1420 cm^−1^ fingerprint region confirms co-existence of diverse nitrogen- and oxygen-containing surface functionalities responsible for excellent aqueous dispersebility.^37,38^

The UV–Vis absorption spectra (Fig. 5) exhibit strong absorption below ∼230 nm, attributed to π → π* transitions of the sp^2^-conjugated carbon framework, together with a shoulder at 273–274 nm in B2-CQD and B3-CQD, along with 253 nm in B3-CQD (Table S5), arising from π → π* transitions associated with C=O/C=N-containing conjugated domains.^41^ The principal n → π* absorption band appears at 342.7 nm (B1-CQD), 328.7 nm (B2-CQD), and 328.0 nm (B3-CQD). This band of citric acid–urea products arises from citrazinic-acid-type species,^16,46^ and aggregation of these species has been shown to shift and broaden it;^42^ the red-shifted and broader band of B1-CQD is consistent with this behavior. B3-CQD further exhibits a maximum at 406.7 nm. However, B3-CQD shows a higher raw absorbance across the spectrum; the normalized absorbance at 410–414 nm is similar for all three samples (Fig. 5b), so this weak absorption is not unique to B3-CQD (for B3-CQD, the normalized values are upper limits, because its absorbance at 200 nm exceeds the linear range of the spectrophotometer).

**Fig. 5:**
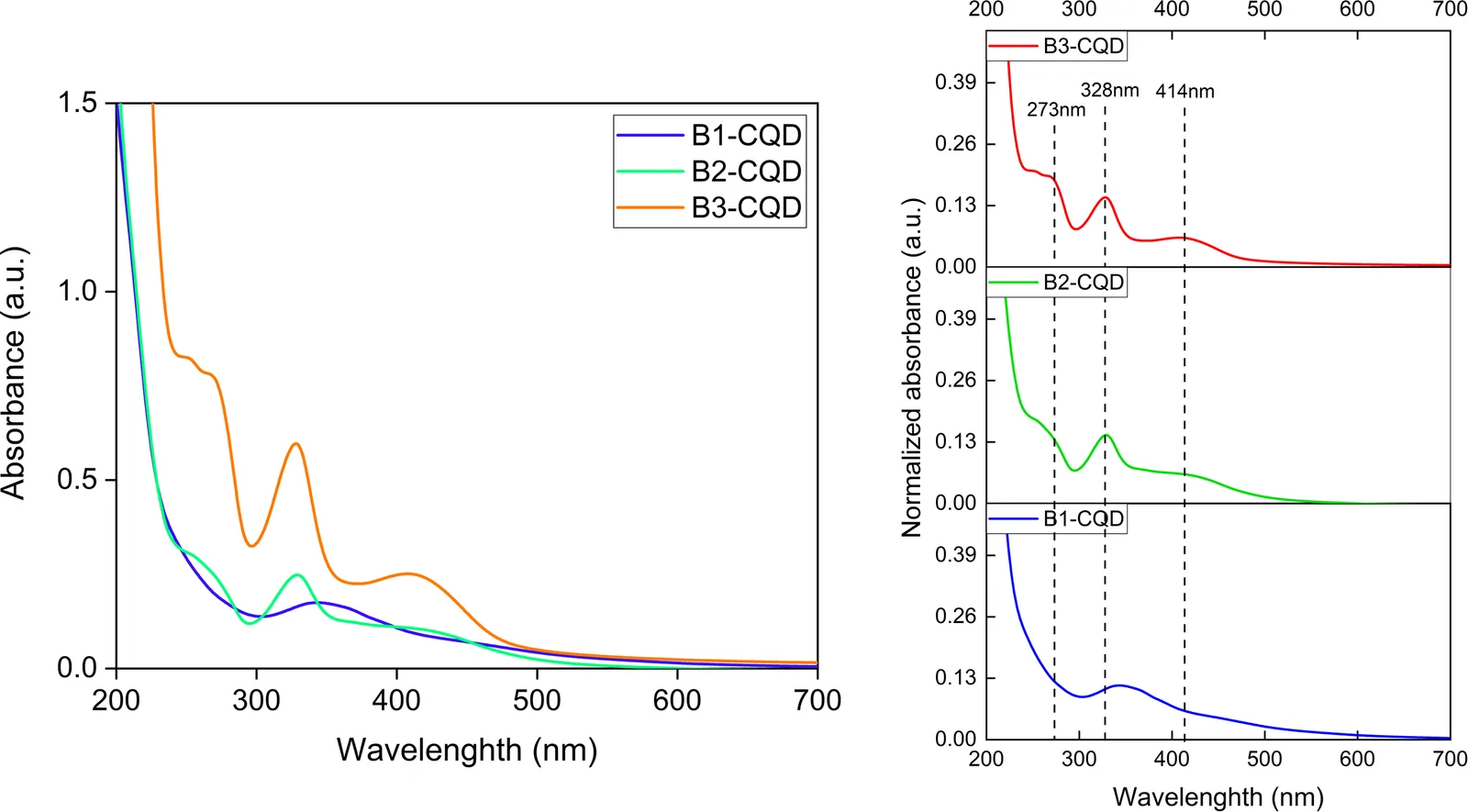
(Left) UV-Vis absorption spectra of B1-CQD (blue), B2-CQD (green), and B3-CQD (orange) in aqueous dispersion over 200–700 nm, showing π → π* and n → π* transitions; absorption maxima and shoulders are listed in Table S5. (Right) Normalized absorbance spectra, obtained by dividing each spectrum by its maximum absorbance (at 200 nm); the ordinate is truncated at 0.39 to show the features of interest. For B3-CQD, the absorbance at 200 nm exceeds the spectrophotometer's linear range, so the normalized values are upper limits.

Optical transition energies determined from Tauc plots (Fig. 6 and Table 6) comprise a core-state (π → π*) gap and sub-gap onsets. The core-state gap was obtained from the linear fit of the single band-edge region, and each sub-gap onset from a single tangent at the steepest point of the corresponding absorption feature (each peak or shoulder, identified as an inflection point on a rising edge).^43^ Only onsets agreeing within 0.06 eV between extrapolation to the energy axis and to the sub-edge baseline^44^ are reported.

**Fig. 6:**
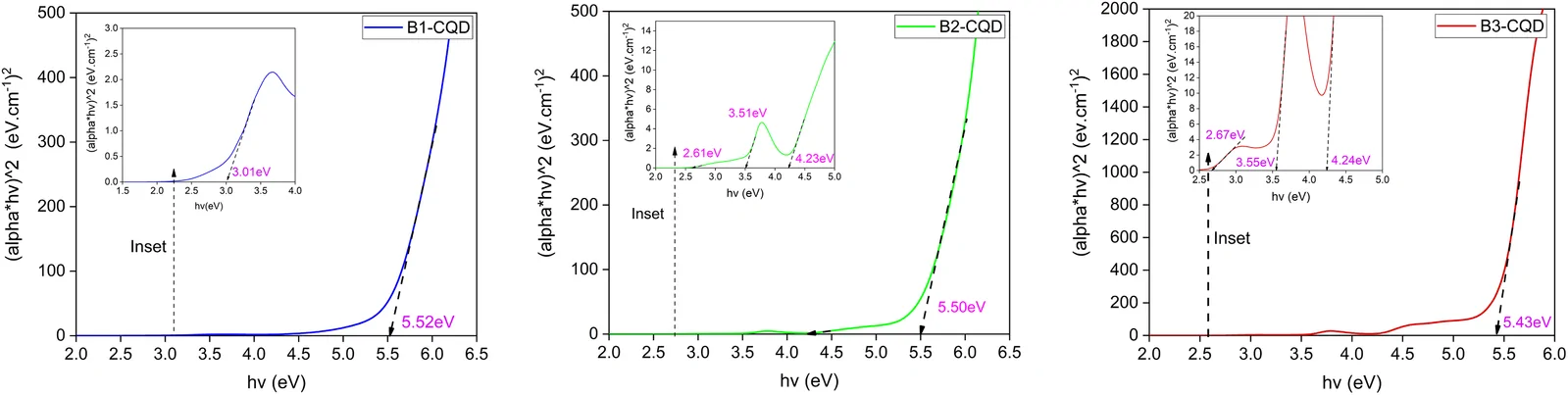
Tauc plots of B1-CQD, B2-CQD, and B3-CQD, measured at the same concentration. Main panels: core-state (π → π*) absorption edge, with the linear fit extrapolated to the energy axis (5.52, 5.50 and 5.43 eV; For B3-CQD, the absorbance at the common measurement concentration exceeded the reliable range of the spectrophotometer in the region used for the core-state Tauc analysis, so its core-state value is indicative only; all other values were obtained within the reliable range). Insets: sub-gap region, with each onset obtained from a single tangent at the steepest point of an absorption feature.

All three samples were measured at the same concentration (0.0332 mg mL^−1^) to allow direct comparison. The core-state gap is 5.52 eV for B1-CQD and 5.50 eV for B2-CQD; at this common concentration, the absorbance of B3-CQD in the core-state region (below ∼230 nm) exceeded the linear range of the spectrophotometer, so its value of 5.43 eV is indicative only, and no trend in the core-state gap is claimed. All other B3-CQD values were obtained within the reliable range. Sub-gap onsets are found at 3.01 eV (B1-CQD), 2.61, 3.51, and 4.23 eV (B2-CQD), and 2.67, 3.55, and 4.24 eV (B3-CQD), associated with nitrogen- and oxygen-containing groups whose type and abundance depend on the precursor composition.^38,45,46^ As particle size and nitrogen content co-vary in this series, their individual contributions to these transitions cannot be separated.

Photoluminescence (PL) spectra recorded at multiple excitation wavelengths (Fig. 7 and Table 7) exhibit pronounced excitation-dependent emission across all three CQD samples, a characteristic feature commonly attributed to multiple emissive surface states and heterogeneous electronic environments in carbon quantum dots.^49,50^ B1-CQD and B2-CQD exhibit maximum emission centered at ∼438 and ∼442 nm, respectively, under 360 nm excitation, whereas B3-CQD displays its maximum emission at ∼370 nm under 320 nm excitation and emits at ∼438 nm under 360 nm excitation, within 3% of its maximum intensity. In each sample, the emission maximum is red-shifted relative to its own excitation wavelength, consistent with Stokes' law; the lower emission wavelength of B3-CQD at its own optimal excitation is a difference between samples. For B1-CQD, the emission maximum remains within ∼425–438 nm for excitation between 300 and 360 nm, consistent with a single dominant emitter over this range; a contribution from co-produced molecular fluorophores cannot be excluded without further purification.

**Fig. 7:**
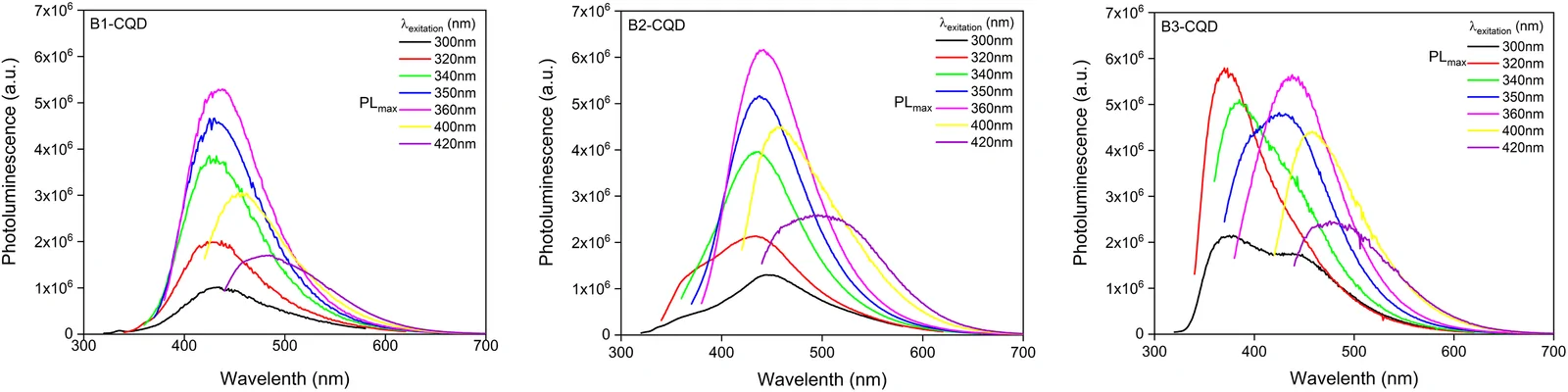
Photoluminescence emission spectra of B1-CQD, B2-CQD, and B3-CQD recorded at multiple excitation wavelengths (280–420 nm; 300–420 nm for B1-CQD); raw intensities are shown, without baseline correction or offset. B3-CQD shows its emission maximum at 370 nm (*λ*_ex_ = 320 nm), and all samples emit at 438–442 nm under 360 nm excitation.

**Table 7:** Photoluminescence parameters for B1-CQD, B2-CQD, and B3-CQD. The tuning range is the range of emission maxima across the excitation wavelengths used

Sample	Optimal *λ*_ex_ (nm)	PL max (nm)	Tuning range (nm)
B1-CQD	360	438	425–485
B2-CQD	360	442	435–492
B3-CQD	320	370	369–476

This distinct emission behavior is consistent with the evolution of nitrogen-induced surface electronic states indicated by XPS and with the sub-gap levels determined from Tauc analysis. PL intensities are relative values recorded under identical conditions. B3-CQD exhibits the widest excitation-tunable emission range (369–476 nm, defined as the range of emission maxima across the excitation wavelengths used; 425–485 nm for B1-CQD and 435–492 nm for B2-CQD), consistent with a wider distribution of emissive states. Collectively, the excitation-dependent emission behavior, together with the UV-Vis absorption, Tauc analysis, and XPS results, is consistent with surface-state-mediated emission, although a contribution from co-produced molecular fluorophores cannot be excluded.^37,38,49–51^

Comparison of the Tauc-derived transition energies with the excitation-dependent PL spectra shows that the emission correlates with the sub-gap levels rather than with the core-state (π → π*) gap. Comparing each sub-gap absorption feature with the emission observed under excitation near that energy gives Stokes shifts of 0.4–0.9 eV (*e.g.*, B1-CQD: 337 nm absorption to 425 nm emission under 340 nm excitation, 0.76 eV), consistent with emission from lower-energy surface-state emissive centers rather than direct core-state band-edge emission.^52^ The emission of B3-CQD depends on which level is excited: under 320 nm excitation (into its 3.79 eV feature), it emits at 370 nm, whereas under 400 nm excitation (into its 3.09 eV feature), it emits at 458 nm. The core-state gap (∼5.4–5.5 eV) lies well above the observed emission energies; the corresponding Stokes shift would exceed 2.5 eV, which is not consistent with a direct relaxation pathway. These observations indicate that absorption and emission involve multiple electronic and surface states, with relaxation among these states preceding radiative recombination.^11^

The comprehensive characterization suggests the following qualitative picture. During microwave carbonization, citric acid undergoes rapid dehydration and cyclization to form the graphitic carbon core, while urea decomposes to generate reactive nitrogen species incorporated as dopants and surface groups. At low urea content (B1), carbon-dominated carbonization yields moderate N-doping (20.5%) and the largest particles (4.79 nm). At intermediate urea content (B2), N-doping increases (24.0%), and the particle size decreases (4.03 nm). At high urea content (B3), excess urea simultaneously acts as a heavy nitrogen dopant (31.9%) and a growth inhibitor, yielding the smallest (2.95 nm), most monodisperse particles. As particle size and nitrogen content co-vary across the series, their individual contributions to the optical properties cannot be separated.

## 4. Conclusions

Nitrogen-doped carbon quantum dots were prepared by rapid microwave carbonization of citric acid and urea at three mass ratios, giving a controlled series in which precursor stoichiometry is the only synthetic variable. Increasing the urea content raised the nitrogen content from 20.5 to 31.9% at a similar total oxygen content, reduced the mean particle size from 4.79 to 2.95 nm, and increased the Raman *I*_D_/*I*_G_ (integrated area) ratio from 1.99 to 2.26. The surface-to-bulk nitrogen ratio (SBNR) increased from 34.1% to 82.4%, indicating that the surface nitrogen content progressively approaches the bulk value (a descriptive ratio, since the XPS sampling depth is comparable to the particle size). At the same time, N 1s XPS indicated a redistribution of nitrogen from pyridinic/amine towards pyrrolic/amide environments.

A distinct evolution of the optical response accompanies these changes. All three samples share the main n → π* absorption band (328–343 nm) and a core-state gap of ∼5.4–5.5 eV, whereas B2-CQD and B3-CQD develop an additional absorption feature at 273–274 nm and sub-gap levels near 4.2 eV. The photoluminescence follows these sub-gap levels rather than the core-state gap, and B3-CQD shows the widest excitation-tunable emission range (369–476 nm). Because the core-state gap is essentially unchanged between B1-CQD and B2-CQD (5.52 and 5.50 eV) despite the decrease in particle size, while new features associated with nitrogen- and oxygen-containing groups emerge, these optical changes are consistent with a predominant role of the nitrogen-rich surface chemistry. However, particle size and nitrogen content vary together in this series.

Precursor stoichiometry, therefore, offers a simple lever for tuning the nitrogen content, surface chemistry, and sub-gap states of citric acid–urea-derived carbon quantum dots, with the SBNR providing a convenient descriptive measure of how the surface and bulk nitrogen contents evolve. These insights can guide the design of carbon quantum dots for sensing, bioimaging, photocatalysis, and optoelectronic applications. Future work will extend this study through dialysis-based purification and identification of the crystalline by-product; replicate synthesis; a nitrogen-free control; depth-resolved XPS; quantum yield and pH-dependent photoluminescence measurements; and time-resolved photoluminescence and theoretical studies of the excited-state dynamics.

## Author contributions

Shweta Dua: conceptualization, methodology, investigation, formal analysis, software, data curation, writing – original draft. Pawan Kumar: formal analysis, writing – review & editing. Sakshi Kapoor: resources. Manoj Kumar Khanna: resources, supervision. Geeta Bhatt: resources, writing – review & editing, supervision, validation.

## Conflicts of interest

The authors declare no conflicts of interest.

## Supplementary Material

RA-OLF-D6RA07516A-s001

## Data Availability

Most of the data obtained and/or analyzed is reported in the manuscript and the supplementary information (SI). The raw datasets used and/or analyzed during the current study are available from the corresponding author on reasonable request. Supplementary information is available. See DOI: https://doi.org/10.1039/d6ra07516a.
