# Supplementary material for: Tuning carbon quantum dot optics through nitrogen surface chemistry
Source: RSC Adv. 2026 Oct 14;16(47):62724–34. doi: 10.1039/d6ra07516a (PMC13662548; doi:10.1039/d6ra07516a)
Supplement: Supplementary file 1 [file RA-016-D6RA07516A-s001.pdf]

Supplementary Information (SI) for RSC Advances.
This journal is © The Royal Society of Chemistry

Supplementary Information

Tuning Carbon Quantum Dot Optics through Nitrogen Surface Chemistry

Fig. S1. Raman Baseline Correction and Band Fitting

Fig. S1

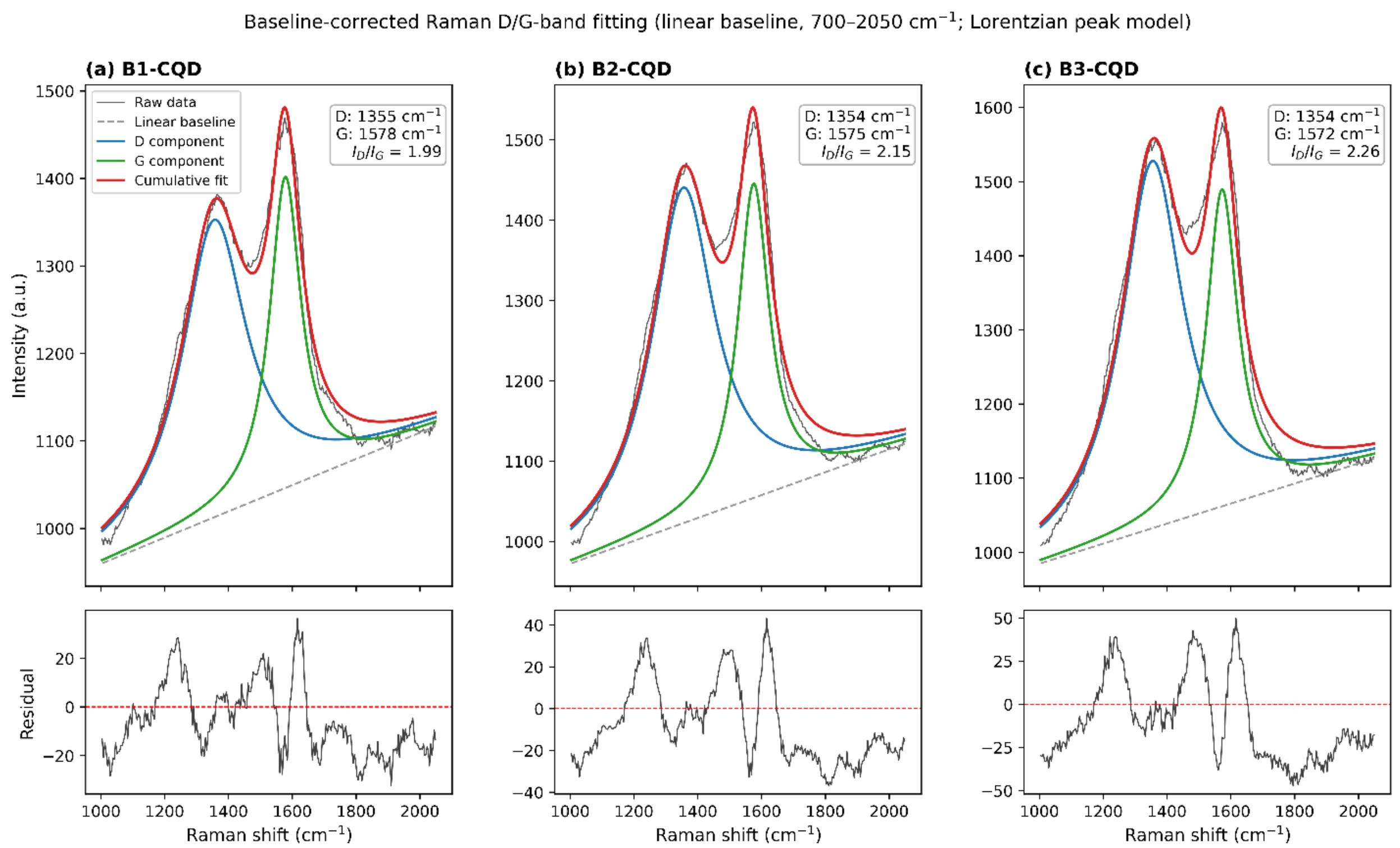

Fig. S1. Baseline-corrected fitting of the Raman D and G bands of (a) B1-CQD, (b) B2-CQD and (c) B3-CQD. A linear baseline (700–2050 cm^{-1}) was subtracted, and the D and G bands were fitted with Lorentzian profiles. Each upper panel shows the raw data, linear baseline, D and G components and cumulative fit; each lower panel shows the fit residual. The fitted positions and integrated-area ratios are D 1355 and G 1578 cm^{-1}, I_D/I_G = 1.99 (B1-CQD); D 1354 and G 1575 cm^{-1}, I_D/I_G = 2.15 (B2-CQD); and D 1354 and G 1572 cm^{-1}, I_D/I_G = 2.26 (B3-CQD).

Fig. S2. Representative annotated TEM fields

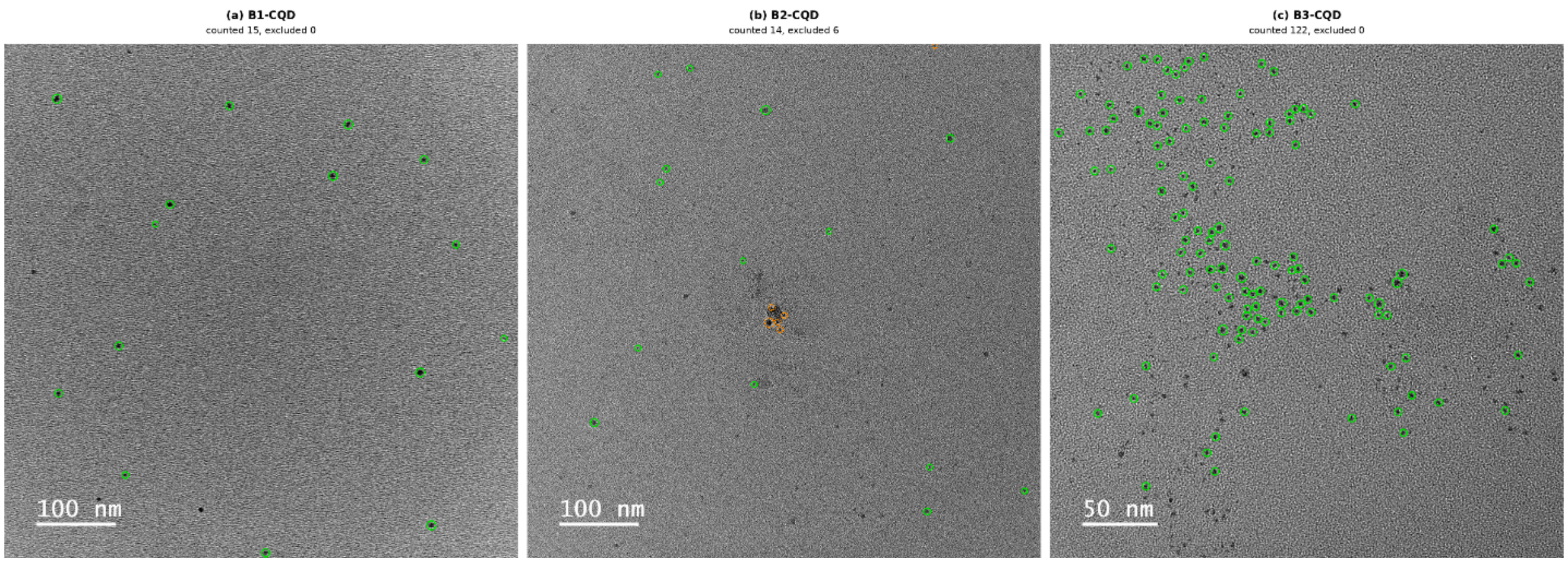

Fig. S2. Representative annotated TEM fields of B1-CQD, B2-CQD and B3-CQD used for particle-size analysis. Green circles mark counted particles; orange dashed circles mark detected particles excluded as fused aggregates or edge-truncated particles. Only features exceeding a uniform contrast threshold, applied identically to all fields, were counted; fainter features, which could not be distinguished reliably from the support-film background, were not counted.

Fig. S3. EDX Bar Chart

Fig. S3

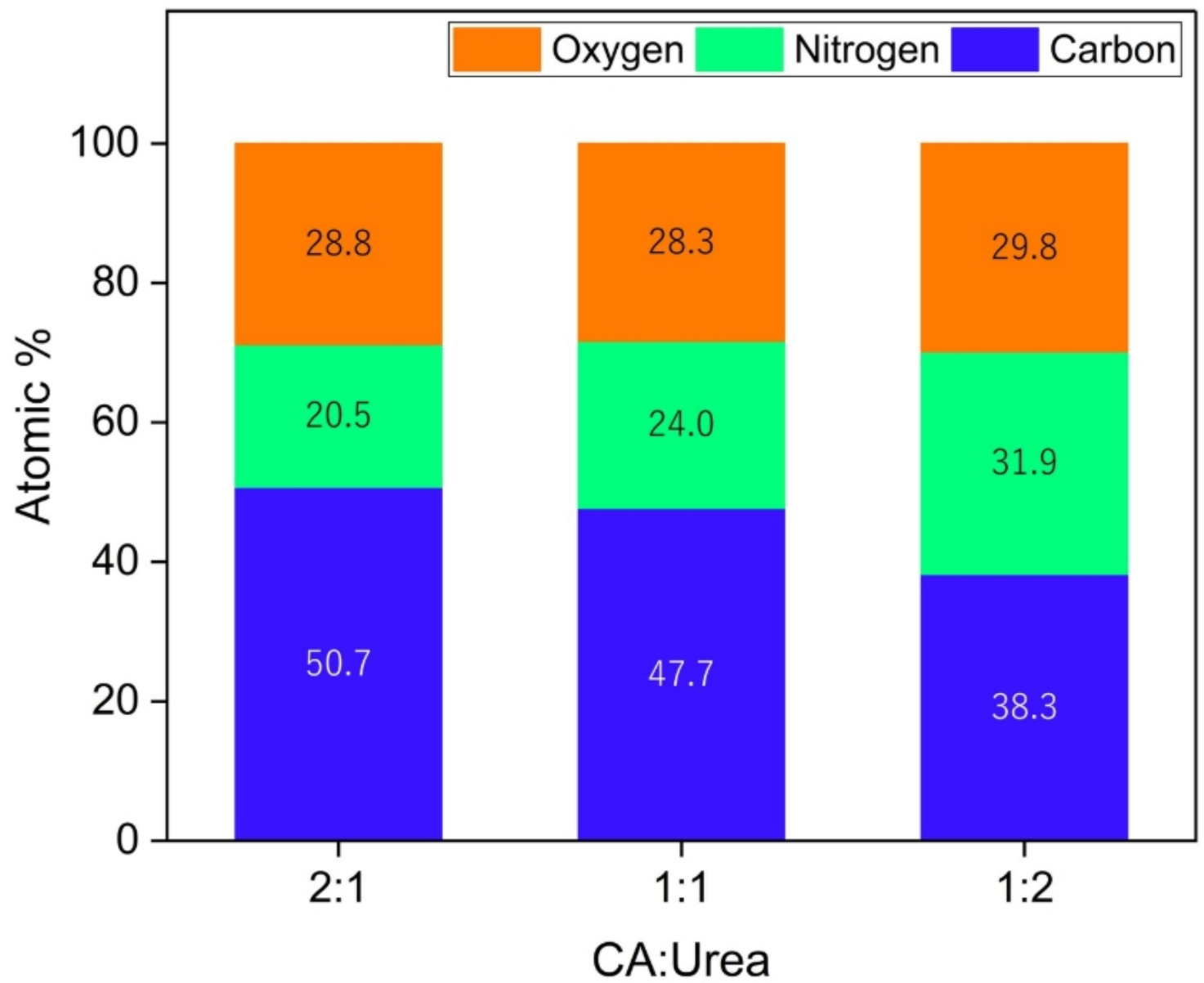

Fig. S3. EDX elemental composition bar chart showing atomic percentages of carbon (blue), nitrogen (green), and oxygen (orange) for B1-CQD (CA:urea mass ratio = 2:1), B2-CQD (1:1), and B3-CQD (1:2), demonstrating systematic increase in nitrogen content with increasing urea ratio. Values exclude a minor aluminium signal (∼0.4%) attributed to the aluminium sample stub; quantifications with and without aluminium are given in Table S2.

Fig. S4. XPS Survey Spectra

Fig. S4

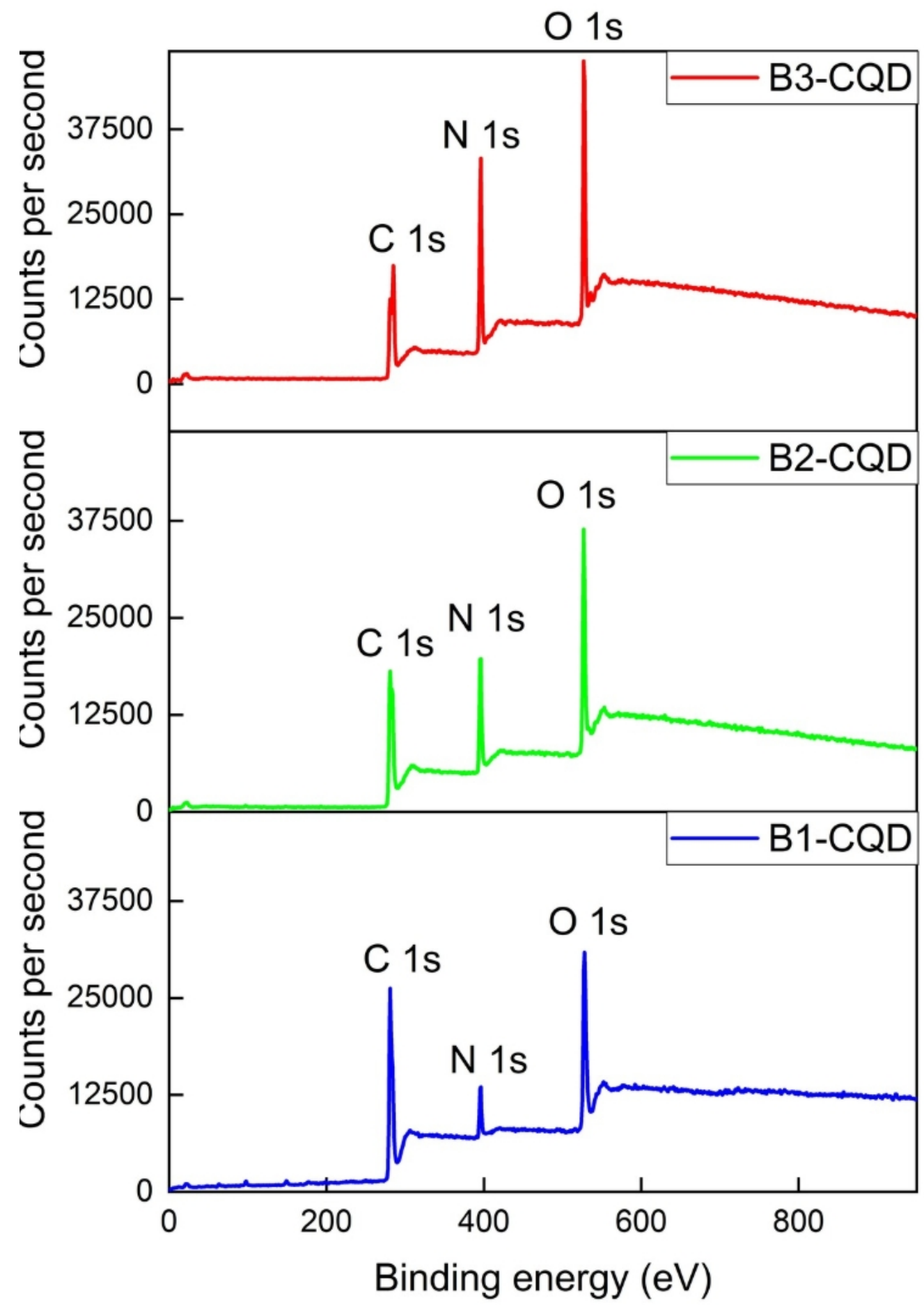

Fig. S4. XPS survey spectra of B1-CQD (blue), B2-CQD (green) and B3-CQD (red) over the binding-energy range 0–900 eV, showing C 1s (∼284 eV), N 1s (∼400 eV) and O 1s (∼532 eV) peaks, with no impurity signals detected within the XPS sampling depth.

Fig. S5. High-Resolution XPS Fits with Raw Data and Residuals

Fig. S5

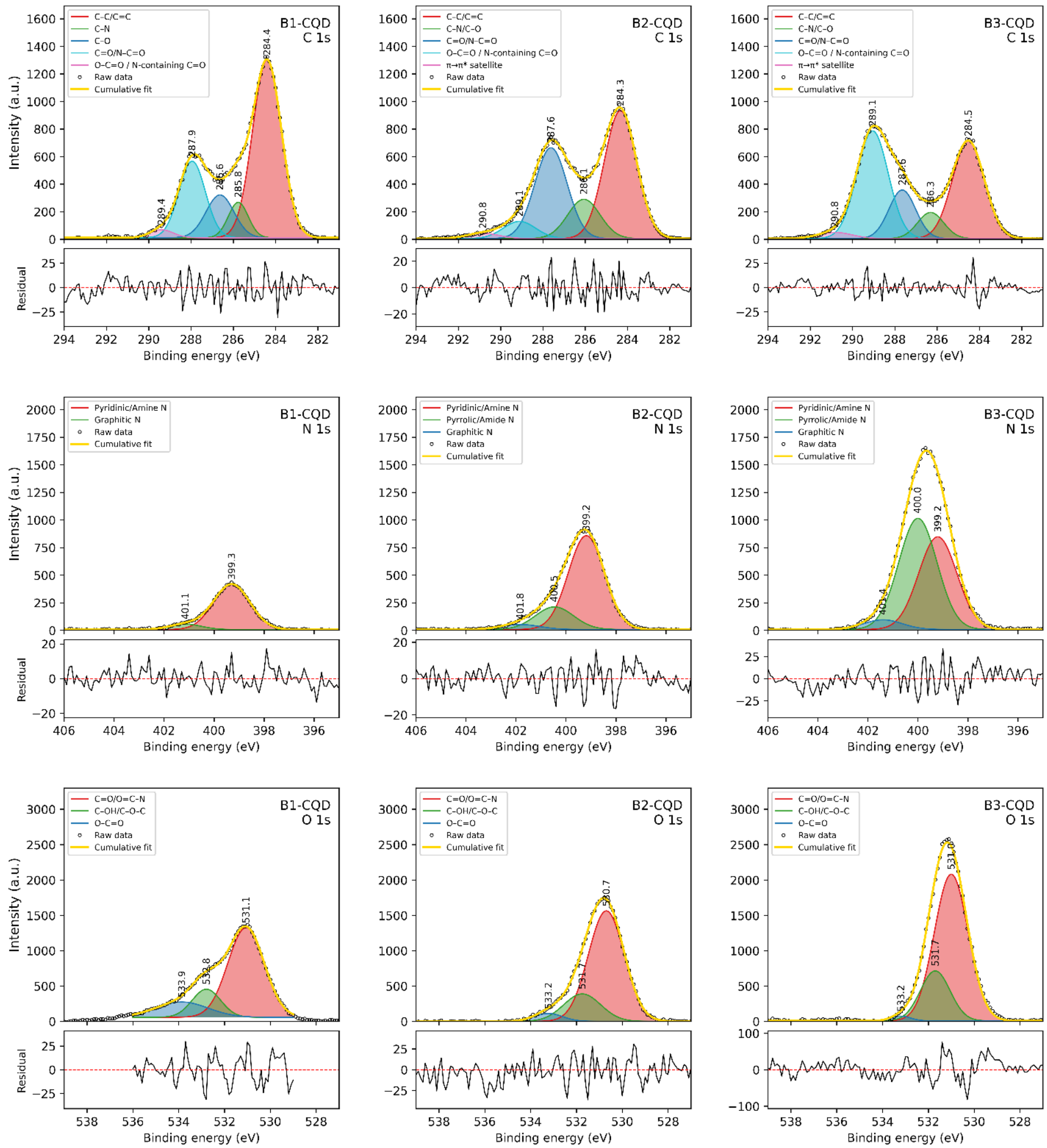

Fig. S5. High-resolution C 1s, N 1s and O 1s XPS spectra of B1-CQD, B2-CQD and B3-CQD, showing the background-subtracted data (open circles), fitted Gaussian components (shaded), cumulative fit and fit residuals (lower panels). The fitted parameters are listed in Table S4.

Fig. S6. FTIR Spectrum of B1-CQD, B2-CQD, and B3-CQD

Fig. S6

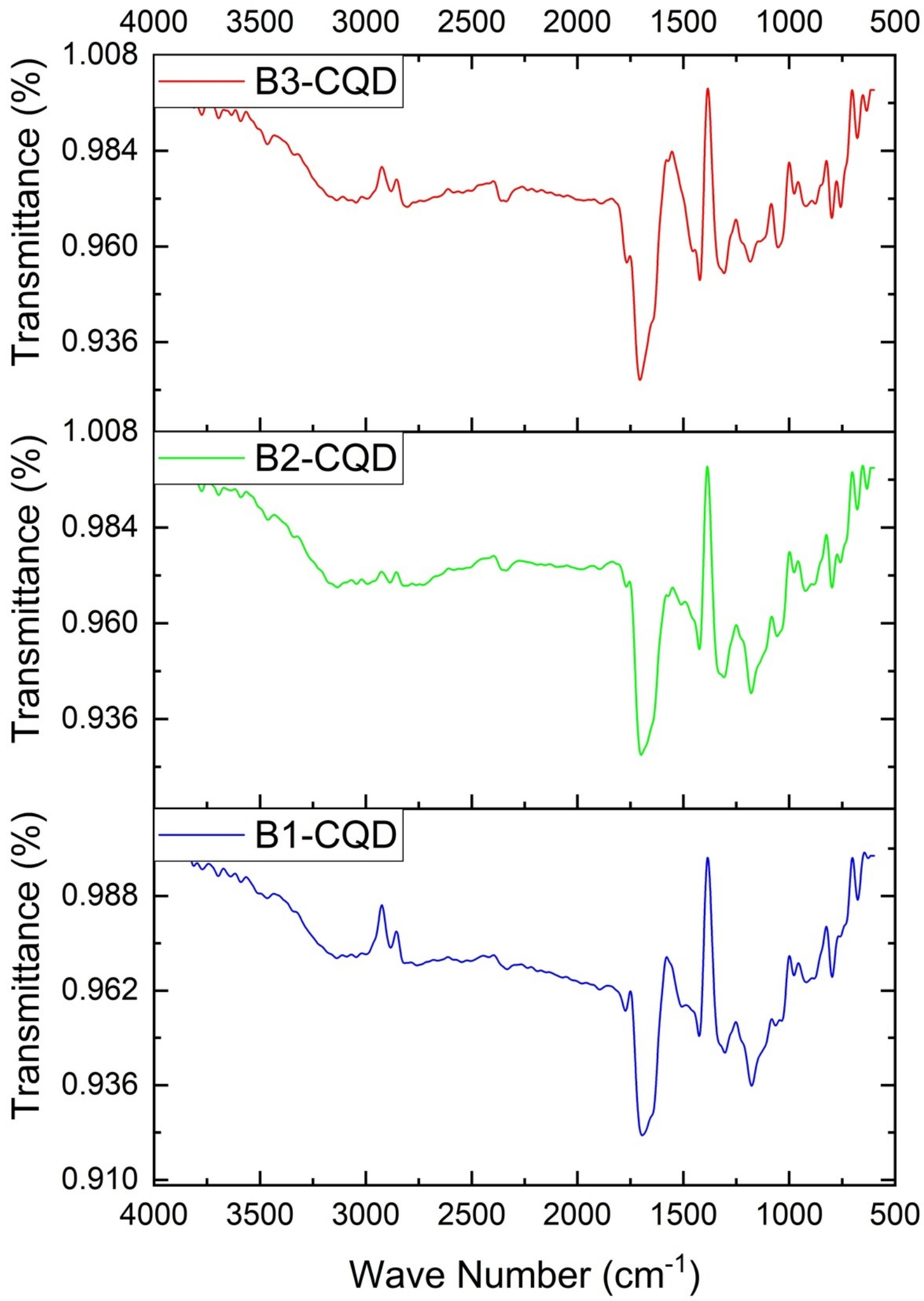

Fig. S6. FTIR spectra of B1-CQD (blue), B2-CQD (green), and B3-CQD (red) in the range 4000–500 cm^{-1}, showing characteristic O–H/N–H, C–H, C=O, C=C/C=N, and C–N/C–O vibrational bands with systematic evolution of N–H and C=N intensities.

Supplementary Tables

Table S1. XRD peak fitting of the (002)-type reflection and the additional reflections of B3-CQD, with Scherrer domain sizes and TEM diameters.

Sample	Reflection	~ 2θ (°)	FWHM (°)	Scherrer size (nm)	TEM, number-average (nm)
B1-CQD	(002)-type	26.84	4.22	1.9	4.79
B2-CQD	(002)-type	27.34	2.73	3.0	4.03
B3-CQD	(002)-type, overlapping by-product	27.53	1.92	4.3*	2.95
	by-product	20.05	0.81	—	—
	by-product, overlapping (002)-type	28.41	0.23-0.29	28-36	—
	by-product, (002)-type	29.7	—	—	—

*Apparent value, affected by overlap with by-product reflections; not attributed to the CQDs

Each reflection was represented by a single Gaussian component with a linear baseline over 2θ = 15–33° (fitted simultaneously for the four reflections of B3-CQD), and Scherrer sizes were calculated with K = 0.9 and λ = 0.1542 nm. 2θ values are fitted centres; for the asymmetric (002)-type reflections these lie below the peak maxima (27.4–27.5°) quoted in the main text. Instrumental broadening and the unresolved Cu $K\alpha_1$/$K\alpha_2$ doublet were not corrected, so the Scherrer sizes are lower bounds. Reflections assigned to crystalline by-products are not attributed to the CQDs. The widths of the 20.05° and ~29.7° reflections depend strongly on the fit and are not reported; for the 28.41° reflection, the range reflects different fit windows and models, and in all cases the domain size far exceeds the particle size.

Table S2. EDX elemental composition of B1-CQD, B2-CQD and B3-CQD, quantified including and excluding the minor aluminium signal.

Sample	Element	Including Al		Excluding Al (reported)	
		Atomic %	Weight % ± σ	Atomic %	Weight % ± σ
B1-CQD	C	51.4	45.37 ± 0.73	50.7	44.91 ± 0.74
	N	20.2	20.79 ± 1.18	20.5	21.13 ± 1.19
	O	28.0	32.99 ± 0.60	28.8	33.95 ± 0.62
	Al	0.42	0.84 ± 0.03	—	—
B2-CQD	C	48.3	42.48 ± 0.72	47.7	42.10 ± 0.71
	N	23.7	24.37 ± 1.15	24.0	24.64 ± 1.14
	O	27.5	32.26 ± 0.62	28.3	33.26 ± 0.63
	Al	0.45	0.89 ± 0.04	—	—
B3-CQD	C	38.8	33.58 ± 0.45	38.3	33.23 ± 0.44

	N	31.7	31.99 ± 0.75	31.9	32.27 ± 0.74
	O	29.1	33.58 ± 0.50	29.8	34.50 ± 0.50
	Al	0.43	0.84 ± 0.03	—	—

One spectrum per sample was recorded from a single field (image width 632.1 μm) at 15 kV with an acquisition time of 89.8–90.3 s, and quantified with the "all elements (normalised)" method. σ is the statistical uncertainty of the weight percentage reported by the instrument software. The aluminium signal (0.42–0.45%, similar in all three samples) is attributed to the aluminium sample stub; the values excluding aluminium are those reported in the main text.

Table S3. XPS quantitative analysis — raw areas, RSF values, RSF-corrected areas, and derived atomic percentages.

Sample	Element	Raw Area	RSF	Area/RSF	Atomic %	N/C ratio
B1-CQD	C 1s	113010	1.00	113010	71.9%	0.097
B1-CQD	N 1s	19867	1.80	11037	7.0%	—
B1-CQD	O 1s	96872	2.93	33062	21.0%	—
B2-CQD	C 1s	96772	1.00	96772	60.7%	0.283
B2-CQD	N 1s	49459	1.80	27477	17.2%	—
B2-CQD	O 1s	102956	2.93	35139	22.0%	—
B3-CQD	C 1s	86655	1.00	86655	49.6%	0.530
B3-CQD	N 1s	82555	1.80	45864	26.3%	—
B3-CQD	O 1s	123437	2.93	42129	24.1%	—

Table S4. XPS peak-fitting parameters of the C 1s, N 1s and O 1s spectra of B1-CQD, B2-CQD and B3-CQD.

Component	Position (eV)	FWHM (eV)	Area (a.u.)	Area (%)
**B1-CQD, C 1s** (R^2 = 0.99919; offset y0 = 10.5 ± 1.9)				
C–C/C=C	284.41 ± 0.02	1.61 ± 0.02	2207 ± 55	55.5 ± 1.4
C–N	285.77 ± 0.47	1.11 ± 0.83	303 ± 1815	7.6 ± 45.7
C–O	286.63 ± 0.86	1.56 ± 5.76	515 ± 3253	13.0 ± 81.9
C=O/N–C=O	287.95 ± 0.73	1.47 ± 0.83	868 ± 1521	21.8 ± 38.3
O–C=O / N-containing C=O	289.39 ± 0.67	1.31 ± 0.62	81 ± 117	2.0 ± 2.9
**B2-CQD, C 1s** (R^2 = 0.99935; offset y0 = 4.8 ± 1.5)				
C–C/C=C	284.34 ± 0.04	1.80‡ ± 0.04	1778 ± 103	45.6 ± 2.6
C–N/C–O	286.06 ± 0.42	1.80‡ ± 0.75	547 ± 524	14.0 ± 13.4

C=O/N–C=O	287.62 ± 0.12	1.80‡ ± 0.62	1263 ± 944	32.4 ± 24.2
O–C=O / N-containing C=O	289.11 ± 1.19	1.80‡ ± 1.89	239 ± 625	6.1 ± 16.0
π→π* satellite	290.84 ± 0.99	1.80‡ ± 0.95	73 ± 107	1.9 ± 2.7
**B3-CQD, C 1s (R² = 0.99934; offset y0 = 4.8 ± 1.5)**				
C–C/C=C	284.52 ± 0.01	1.78 ± 0.02	1345 ± 25	36.0 ± 0.7
C–N/C–O	286.31 ± 0.18	1.46 ± 0.29	294 ± 126	7.9 ± 3.4
C=O/N–C=O	287.65 ± 0.08	1.49 ± 0.32	558 ± 270	14.9 ± 7.2
O–C=O / N-containing C=O	289.05 ± 0.06	1.74 ± 0.16	1450 ± 231	38.8 ± 6.2
π→π* satellite	290.84 ± 0.59	1.90‡ ± 0.67	91 ± 69	2.4 ± 1.9
**B1-CQD, N 1s (R² = 0.9982; offset y0 = 6.8 ± 0.8)**				
Pyridinic/Amine N	399.29 ± 0.01	1.70 ± 0.02	747 ± 9	90.5 ± 1.0
Graphitic N	401.14 ± 0.07	1.51 ± 0.13	78 ± 8	9.5 ± 1.0
**B2-CQD, N 1s (R² = 0.9995; offset y0 = 7.7 ± 1.2)**				
Pyridinic/Amine N	399.19 ± 0.10	1.70 ± 0.06	1535 ± 331	76.2 ± 16.4
Pyrrolic/Amide N	400.46 ± 0.24	1.80‡ ± 1.25	393 ± 613	19.5 ± 30.5
Graphitic N	401.82 ± 1.97	1.80‡ ± 1.35	86 ± 302	4.3 ± 15.0
**B3-CQD, N 1s (R² = 0.99944; offset y0 = 7.7 ± 2.4)**				
Pyridinic/Amine N	399.21 ± 2.26	1.80‡ ± 0.82	1609 ± 10384	43.4 ± 280.2
Pyrrolic/Amide N	400.00 ± 2.04	1.80‡ ± 1.73	1929 ± 11200	52.0 ± 302.2
Graphitic N	401.40 ± 2.85	1.80‡ ± 1.79	168 ± 886	4.5 ± 23.9
**B1-CQD, O 1s (R² = 0.99882; offset y0 = 58.4 ± 11.3)**				
C=O/O=C–N	531.06 ± 0.02	1.87 ± 0.03	2518 ± 73	67.0 ± 1.9
C–OH/C–O–C	532.79 ± 0.03	1.47 ± 0.18	620 ± 317	16.5 ± 8.4
O–C=O	533.87 ± 0.71	2.70 ± 0.83	622 ± 401	16.5 ± 10.7
**B2-CQD, O 1s (R² = 0.99944; offset y0 = 1.2 ± 4.0)**				
C=O/O=C–N	530.70 ± 0.55	1.84 ± 0.25	3059 ± 4106	76.2 ± 102.2
C–OH/C–O–C	531.75 ± 2.70	1.90‡ ± 3.37	785 ± 4525	19.5 ± 112.7
O–C=O	533.23 ± 0.77	1.47 ± 0.62	173 ± 457	4.3 ± 11.4
**B3-CQD, O 1s (R² = 0.99896; offset y0 = 8.3 ± 4.1)**				
C=O/O=C–N	531.00‡ ± 0.71	1.73 ± 0.37	3825 ± 5481	75.8 ± 108.7
C–OH/C–O–C	531.70‡ ± 0.91	1.53 ± 0.73	1147 ± 5522	22.8 ± 109.5
O–C=O	533.20‡ ± 0.29	1.00‡ ± 0.46	71 ± 67	1.4 ± 1.3

Spectra were charge-referenced to the C–C/C=C component at 284.4 eV and fitted with Gaussian components (Origin) after Tougaard background subtraction; y_0 is the common constant offset of each fit. Uncertainties are the standard errors reported by Origin. Area percentages are relative to the total fitted area of each spectrum; their uncertainties were estimated from the relative uncertainty of each component area, the total area being fixed by the measured envelope. ‡ Parameter constrained during fitting (fixed or bounded) to stabilise the fit of strongly overlapping components. Large relative uncertainties indicate components that are not independently resolved within the envelope; such components are not interpreted individually in the main text.

Table S5. UV–Vis absorption maxima and shoulder positions of B1-CQD, B2-CQD and B3-CQD.

Sample	Absorption maxima (nm)	Shoulders (nm)
B1-CQD	342.7	—
B2-CQD	328.7	273
B3-CQD	328.0; 406.7	253; 274

Spectra were recorded in aqueous dispersion (0.0332 mg mL^{-1}). Maxima were read from the measured spectra; shoulders were located from Savitzky–Golay second-derivative spectra and are listed only where they are also resolved in the measured spectra.
